# High Quantum Efficiency Rare-Earth-Doped Gd_2_O_2_S:Tb, F Scintillators for Cold Neutron Imaging

**DOI:** 10.3390/molecules28041815

**Published:** 2023-02-15

**Authors:** Bin Tang, Wei Yin, Qibiao Wang, Long Chen, Heyong Huo, Yang Wu, Hongchao Yang, Chenghua Sun, Shuyun Zhou

**Affiliations:** 1 Institute of Nuclear Physics and Chemistry, China Academy of Engineering Physics, Mianyang 621000, China; 2 School of Computer Science and Engineering, Sichuan University of Science & Engineering, Zigong 643000, China; 3 Key Laboratory of Photochemical Conversion and Optoelectronic Materials, Technical Institute of Physics and Chemistry, Chinese Academy of Sciences, Beijing 100190, China

**Keywords:** scintillator, cold neutron radiograph, Gd_2_O_2_S:Tb, F

## Abstract

High-resolution neutron radiography provides novel and stirring opportunities to investigate the structures of light elements encased by heavy elements. For this study, a series of Gd_2_O_2_S:Tb, F particles were prepared using a high-temperature solid phase method and then used as a scintillation screen. Upon reaching 293 nm excitation, a bright green emission originated from the Tb^3+^ luminescence center. The level of F doping affected the fluorescence intensity. When the F doping level was 8 mol%, the fluorescence intensity was at its highest. The absolute quantum yield of the synthesized particles reached as high as 77.21%. Gd_2_O_2_S:Tb, F particles were applied to the scintillation screen, showing a resolution on the neutron radiograph as high as 12 μm. These results suggest that the highly efficient Gd_2_O_2_S:Tb, F particles are promising scintillators for the purposes of cold neutron radiography.

## 1. Introduction

Neutron radiography is a powerful tool for detecting soft substances embedded within heavier shells [1], which makes up for the fact that X-ray imaging cannot penetrate such heavy elements. However, compared with X-ray imaging, neutron radiography has more difficulty achieving a sub-10 μm spatial resolution [2]. So far, the limited spatial resolution has been one of the major drawbacks of neutron radiography. In order to conduct nondestructive testing on submicron structures within heavy elements (such as for fuel cell water distribution [3], or the internal structures of culturally significant objects [4], or the water erosion of carbonated mortar [5], etc.) an urgent demand for higher spatial resolution has arisen. Many researchers have improved spatial resolution using different strategies, such as a modification to the structure of a detector or developing novel scintillators.

Some scientists tried to improve the spatial resolution by means of modification of the radiography instrument. Kim [6] proposed the strategy of integrating the recoil proton producing plate and the scintillation plate, and thus improving the imaging resolution by eliminating the process required for the recoil protons to migrate from one plate to another. However, this was only a research suggestion, and no further results were reported. Trtik [7] utilized the opposite approach to improve spatial resolution. They separated the hydrogenous converter material from the scintillator screen, with the expectation that only recoil protons could then reach the scintillation material to produce the final image, and thus the characteristic blurring would be considerably reduced. The results implied a loss of efficiency by around a factor of three but a gain in resolution of up to a factor of two. Some scientists [8,9,10] utilized a magnifying system to enhance the spatial resolution, which achieved 2 μm, with the magnification effect achieved using a microscope system.

In addition to the structure of a radiograph system, the detectors are one of the key factors that influence imaging resolution. Fujine [11] summarized different types of detectors for fast neutron radiography in Japan. The spatial resolutions were 2.5 mm and 1.5 mm for the SIT and CCD camera methods, respectively, while the resolution was 1 mm when using an imaging plate. Bin [12] and Huo [13] reported the performance of the cold neutron radiography beamline, in which a spatial resolution of about 20 μm was obtained when utilizing a Tb-doped Gd_2_O_2_S (GOS) screen with a thickness of 10 μm. Jiang [14] developed a detector prototype; the resolution of the GOS scintillation can be evaluated using neutron beam tests, and a spatial resolution of 36 μm (28 lp/mm) could be measured. The GOS scintillator was supplied by the Paul Scherrer Institute, and had a thickness of 10 µm.

Scintillators are the key materials in scintillation detectors, and the Gd_2_O_2_S:Tb (GOS:Tb) scintillators are primarily used and intensively studied for improving spatial resolution. In order to meet the needs of fuel cell research, Tötzke [3] designed a new type of detector system. The thickness of their optimized GOS scintillator was 5 μm and it achieved a maximum spatial resolution of 25 μm. R. Yasuda [15] prepared scintillation screens using Gd_2_O_2_S:Tb with three different particle sizes and studied the effects of particle size and scintillator thickness on the resolution of neutron radiographs. The results showed that the full width at half maximum (FWHM) values of the signal peaks decreased as the particles’ sizes decreased, and that reducing the thickness of the layers contributed to a high imaging resolution. The best FWHW was over 0.2 mm when the median particle size was about 11.4 μm.

For powder scintillation screens, the minimum thickness of the scintillation screen is determined by the size of powder particles, which affects the resolution. In imaging systems, it has been proposed that nanometer-sized particles would evidently improve the resolution [16,17,18]. Currently, the average particle size of commercial GOS:Tb scintillators is about 3.5 μm, and particles of this size cannot be prepared into thin, tightly packed scintillation screens. Xia [19] prepared GOS:Tb with an average particle size of 20 nm and aggregate particle size of 5–30 μm using a combustion method. It was difficult to control the particle size and dispersion of the sample due to the intense reaction of combustion methods. Nanoparticles with good monodispersity will help to realize their self-assembly into the form of highly ordered patterns, which is more conducive to the preparation of these devices. Therefore, the monodispersity of nanoparticles is very important. Using oleic acid, 1-octadecene, and oleylamine as mixed solvents, Lei [20] innovatively prepared rare-earth-doped GOS nanocrystals with a particle size of 7 nm. However, the sample synthesized using this method had a poor luminescence performance and the quantum efficiency was only 12.9%. Tian [16] prepared a GOS:Tb scintillator using a composite precipitation method (the precipitants were ammonia and ammonium bicarbonate), and the average particle size of the obtained samples was 30–70 nm. The sample prepared using this method had poor monodispersity and particle aggregation. Therefore, it can be said that the efficient synthesis of GOS nanoparticles with high purity is quite challenging. A high-temperature solid phase method is an efficient and rapid synthesis method, which has been widely explored for the preparation of scintillators. Xing [21] prepared spherical precursors with an average diameter of 110 nm. After further calcination and sulphuration, the sample size was reduced to 70–80 nm and showed excellent luminescent properties; however, the spatial resolution value was not mentioned. In recent years, thin powder scintillator screens with traces of isotopically enriched material (^157^GOS:Tb) have been introduced to enhance the light output and absorption in cold neutron radiography. Trtik [22] prepared scintillators using ^157^GOS:Tb with a powder size of 2 μm and layer thicknesses of 2.5 μm; as a result, a spatial resolution of sub-10 μm could be realized. Jan Crha [23] reported that a ^157^GOS scintillation screen for high-resolution neutron radiographs achieved a light yield of 65% (compared to an uncoated scintillation screen) by adding an iridium layer, while maintaining a spatial resolution of 5.2 μm (compared to 4.9 μm for the uncoated screen). In previous work, we [24] proposed a two-step method to fabricate a series of NaF-doped Gd_2_O_2_S:Tb^3+^ (GOS:Tb, F) scintillators. This preparation method can adjust the particle size to a certain extent, and the scintillator can be constructed as an ultra-thin screen, which can achieve a spatial resolution of 12 μm when the size of the scintillator particles is about 420 nm. To improve the resolution, we needed to create a thinner scintillation screen. However, a reduction in scintillation screen thickness would inevitably reduce the neutron detection efficiency. In order to maintain or improve the neutron detection efficiency, it is necessary to improve the luminous intensity of the fluorescent materials accordingly.

In this paper, we synthesized GOS:Tb, F in both micrometer and nanometer grains for use as a scintillator, and did so using a simple high-temperature solid phase method. The effects of NaF on the light yield were intensively studied. The subsequent synthesis of the GOS:Tb, F scintillator demonstrated a highly efficient absolute quantum yield (77.21%) under a 293 nm excitation. Based on this, the thickness of the scintillation screen could be reduced to 2.5 μm. Scintillation screens based on the as-prepared GOS:Tb, F were then applied in a cold neutron radiograph system. The best resolution reached 12 μm, demonstrating the great potential of this scintillation screen for cold neutron radiography applications.

## 2. Results

**Figure 1:**
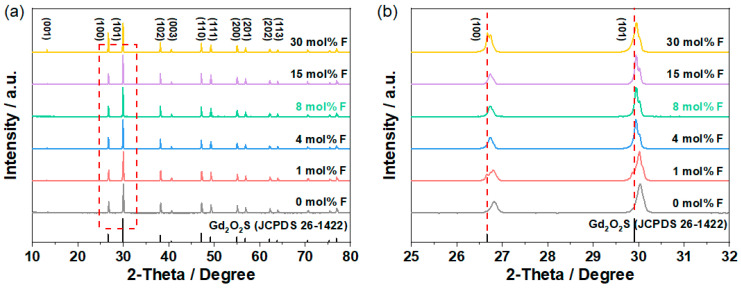
(**a**) XRD profiles and (**b**) local magnified view of the GOS:Tb, *x*F scintillators.

Figure 1a shows the XRD profiles of the GOS:Tb, *x*F scintillators. Obviously, the obtained samples have similar diffraction spectra, and the location of their respective diffraction peaks is consistent with the standard GOS pattern (JCPDS card no. 26-1422), which demonstrates that the GOS:Tb, *x*F scintillators have a hexagonal phase. Furthermore, based on the local magnified view shown in Figure 1b (taking the two main peaks of (100) and (101) as examples), it can be clearly seen that the angles of all the diffraction peaks have slightly shifted in accordance with Bragg’s law: 2dsin θ = nλ, where d is the crystal plane spacing, θ is the angle between the incident ray or the reflected ray and the reflected crystal plane, λ is the wavelength and n is an integer. When F^−^ ions are not doped, because the radius of Tb^3+^ ions (0.177 nm) is smaller than that of Gd^3+^ ions (0.179 nm), lattice shrinkage occurs; d decreases and the angle of the diffraction peak shifts to the high value, indicating that Tb^3+^ ions are successfully doped. When F^−^ ions are doped, lattice expansion is generated because the F^−^ ion radius (0.133 nm) is larger than the O^2−^ ion radius (0.132 nm), and the diffraction angle shifts to a low value, indicating that F^−^ ions are successfully doped [25]. The GOS host lattices are efficiently doped with the use of Tb^3+^ and F^−^ ions.

**Figure 2:**
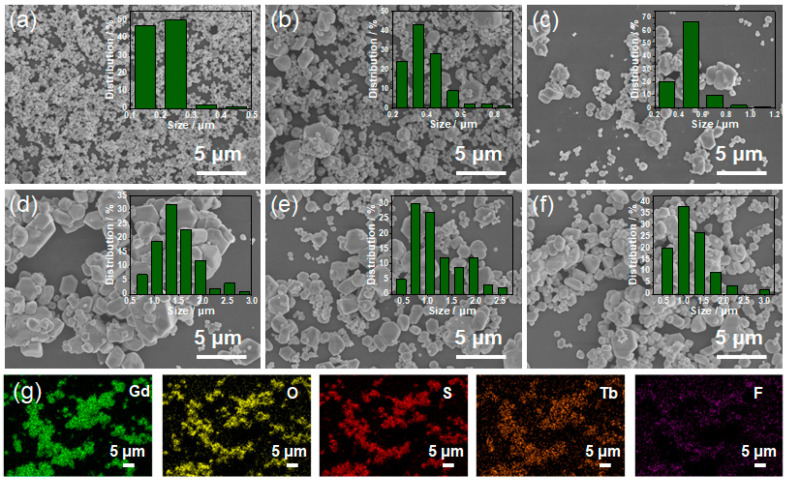
SEM images of GOS:Tb, *x*F scintillators as a function of F levels: (**a**) *x* = 0, (**b**) *x* = 0.01, (**c**) *x* = 0.04, (**d**) *x* = 0.08, (**e**) *x* = 0.15, (**f**) *x* = 0.3. (**g**) EDS element distribution results of the GOS:Tb, 0.08F scintillators.

The shape and size of scintillator particles influence their luminescence characteristics [26]. Figure 2a–f exhibit the SEM results of the GOS:Tb, *x*F scintillators with changing F doping levels. It can be seen that with the increase in F doping levels from 0.01 mol to 0.3 mol, the particle size of the scintillator also changes correspondingly. This indicates that the morphology of scintillation can be regulated using the F doping level. In order to observe the element distribution of the GOS:Tb, *x*F scintillators clearly, an EDS element distribution test was carried out. The results revealed that Gd, O, S, Tb and F elements were uniformly distributed in the particles. Moreover, the Tb and F element were effectively doped into the host lattices.

**Figure 3:**
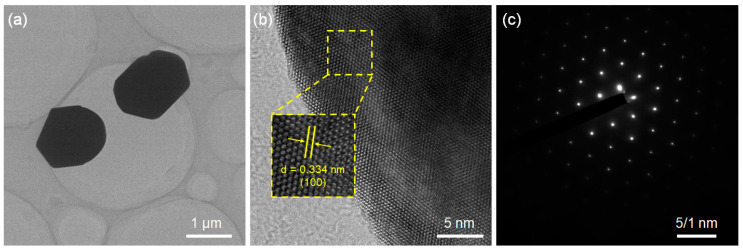
Results of the GOS:Tb, 0.08F scintillators. (**a**) TEM image, (**b**) HR-TEM image and (**c**) SAED pattern.

In order to further study the morphologic characteristics of a GOS:Tb, 0.08F scintillator, a TEM analysis was performed. Figure 3a shows that the GOS:Tb, 0.08F scintillators were of micron scale and irregular in shape. At the same time, the lattice fringe of the GOS:Tb, 0.08F scintillator was studied using high-resolution TEM (HR-TEM, Figure 3b), and the interplanar spacing was calculated to be about 0.334 nm. This was consistent with the (100) interplanar spacing of GOS, proving that the diffraction fringe in the Figure 3b was (100) crystal face. The Selective Area Electron Diffraction (SAED) pattern shown in Figure 3c contains clear bright spots, which demonstrates that the GOS:Tb, 0.08F scintillator has a single-crystal nature [27].

**Figure 4:**
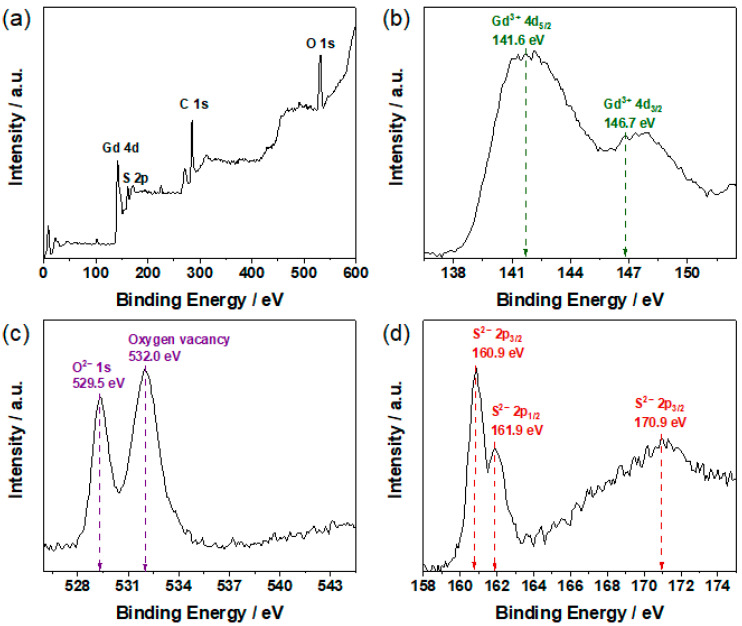
XPS results for the GOS:Tb, 0.08F scintillators. (**a**) XPS survey spectrum, (**b**) Gd^3+^ 4d, (**c**) O^2−^ 1s, (**d**) S^2−^ 2p.

In order to confirm the composition and chemical valence of Gd, O and S in the scintillators, XPS analyses were performed. Figure 4a shows the XPS survey spectrum of the GOS:Tb, 0.08F scintillators, and the presence of Gd, O and S elements can be seen from the corresponding binding energy. As can be seen in Figure 4b, there were two obvious peaks in the Gd^3+^ 4d signal, whose binding energies were 141.6 eV and 146.7 eV respectively, corresponding to Gd^3+^ 4d_5/2_ and Gd^3+^ 4d_3/2_ [28]. Figure 4c depicts the O 1s spectrum. The characteristic peak of binding energy at 529.5 eV can be attributed to O 1s, while the characteristic peak of binding energy at 532.0 eV can be ascribed to oxygen vacancy [29]. In addition, Figure 4d shows the S 2p spectrum, and the characteristic peaks of binding energy at 160.9 eV, 161.9 eV and 170.9 eV were S 2p_3/2_, S 2p_1/2_ and S 2p_3/2_, respectively [30,31]. Therefore, the presence of the Gd^3+^, O^2−^ and S^2−^ ions in the synthesized scintillator was confirmed.

**Figure 5:**
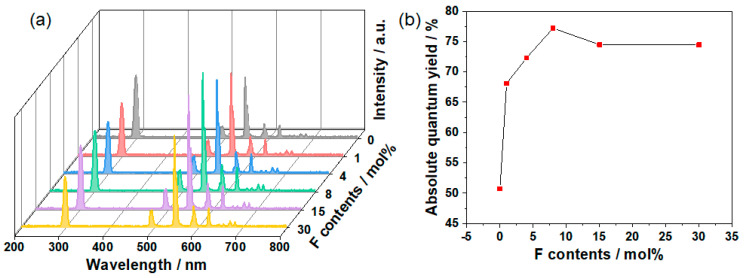
(**a**) Excitation spectra (λ_em_ = 545 nm, 200–400 nm) and emission spectra (λ_ex_ = 298 nm, 400–800 nm) of the GOS:Tb, *x*F scintillators. (**b**) Absolute QY of the GOS:Tb, *x*F scintillators.

The excitation spectra and emission spectra of GOS:Tb, *x*F scintillators with respective dopant levels of 0, 1, 4, 8, 15 and 30 mol% are displayed in Figure 5a. As presented in Figure 5a, when the emission wavelength was 545 nm, the excitation spectra showed an excitation peak (λ_ex_ = 298 nm) between 200 and 400 nm, which was derived from the 4f–5d transitions of Gd^3+^ [32]. Consequently, the emission spectra for GOS:Tb, *x*F scintillators were measured under light excitation levels of 298 nm (Figure 5a). In addition, a series of emission peaks appeared in the emission spectrum range of 400–800 nm. These emission peaks were mainly located at 494 nm, 545 nm, 589 nm, 623 nm, 671 nm and 683 nm, corresponding to the ^5^D_4_→^7^F_6_, ^5^D_4_→^7^F_5_, ^5^D_4_→^7^F_4_, ^5^D_4_→^7^F_3_, ^5^D_4_→^7^F_1_ and ^5^D_4_→^7^F_0_ electronic transition, respectively. These emission spectra indicate that the GOS:Tb, *x*F scintillators have similar emission peaks, among which the strongest emission peak was 545 nm, which can be attributed to the ^5^D_4_→^7^F_5_ transition of Tb^3+^ [33]. Furthermore, it is clear that the F doping level had an effect on emission intensity. With the increase in the F doping level, the emission intensity of GOS:Tb, F scintillators showed a trend of increasing first and then decreasing. When the doping level of F was 8 mol%, the emission intensity was at its best. Due to the concentration quenching effect, the emission intensity decreased when the doping level increased further. The absolute quantum yields (QY) of the resultant scintillators were determined and shown in Figure 5b (λ_ex_ = 298 nm). Consequently, the QY of F in GOS:Tb, *x*F scintillators were revealed to be approximately 50.72, 68.1, 72.31, 77.21, 74.49, 74.47, when the F levels were 0, 1, 4, 8, 15 and 30 mol%, respectively. It is evident that the highest emission intensity was an F doping amount of 8 mol%, which is in agreement with the emission spectra results. The doping of F^-^ ions can generate impurity levels above the top of the valence band when composed of p orbital of oxygen [34]. These impurity levels can be combined with excited electrons to become hole traps to generate fluorescence, and thus improve the luminosity intensity. The replacement of O^2−^ with a certain amount of F^−^ ions could improve the energy transfer efficiency from the GOS host material to Tb^3+^, and contribute to the resulting Tb^3+^ fluorescent transition [35,36]. However, with the increase in the amount of F^−^ ions, new intrinsic defects will be formed, which will lead to a reduction in the energy transfer efficiency and emission intensity [37].

The emission spectra result demonstrate that F^−^ ions had an important effect on the luminescence of scintillators. In order to further investigate the energy transfer between F^−^ and Tb^3+^ ions, the fluorescence lifetimes of GOS:Tb, *x*F scintillators were measured. The fluorescence lifetime was fitted using a single exponential attenuation equation; the formula is as follows [38]:

$$I\left(t\right)=I_{0}+A_{1}\exp\left(-\frac{t}{\tau_{1}}\right)+A_{2}\exp\left(-\frac{t}{\tau_{2}}\right)$$

In this equation, *I*_0_ is the initial emission intensity; *I*(*t*) is the emission intensity at time *t*; *τ*_1_ and *τ*_2_ are the fast and slow attenuation constants of exponential components, respectively; and *A*_1_ and *A*_2_ are the fitting parameters. Consequently, the emission lifetimes of F^−^ in GOS:Tb, *x*F scintillators were revealed to be approximately 638, 598, 590, 584, 586 and 592 μs when the F^−^ levels were 0, 1, 4, 8, 15 and 30 mol%, respectively (Table 1). The fluorescence lifetime of Tb^3+^ noticeably fluctuated in the range of 638–584 μs alongside the increase in the F^−^ ion doping level, indicating that an energy transfer occurs between F^−^ ion and Tb^3+^ ion.

**Figure 6:**
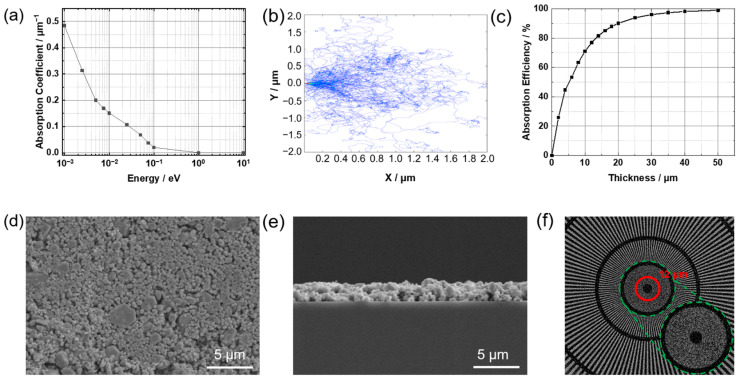
(**a**) Absorption coefficient of the GOS:Tb scintillation screen under different energy neutrons. (**b**) The track of the internal conversion electron (Energy: 38.68 keV) in the scintillation screen. (**c**) The neutron absorption efficiency of scintillation screens of different thicknesses. SEM photographs of the GOS:Tb, F scintillation screen prepared with PVB, (**d**) surface morphology, (**e**) cross-sectional view. (**f**) Results of resolution tests using gadolinium Siemens star.

Geant4 software was used to calculate the neutron absorption coefficient of GOS:Tb for different energies. The physical model for simulation is shown in Figure S1. The physical model used was FTFP_BERT_HP, the neutron source size was 2 cm × 2 cm and the distance was 2 cm from the scintillation screen. The mass ratio of elements in fluorescent screen materials is exhibited in Table S1. Figure 6a shows that the attenuation coefficient reached its highest level at around 10^−3^ eV, and the absorption coefficient decreased with the increase in neutron energy. This is because Gd in GOS:Tb has a large absorption cross section for cold neutrons and generates internal conversion electrons, thus exciting Tb to emit light. The track information of the internal conversion electrons generated by the reaction of neutrons with Gd in the scintillation screen was collected. The tracks of 100 internally converted electrons, with energy of 38.68 keV when incident on the scintillation screen, were also calculated, and the maximum range was about 2.871 μm. Furthermore, the absorption efficiency of cold neutrons in relation to the scintillation screen thickness was calculated. The cold neutron particle source with an emission quantity of 2 × 10^7^ and energy (as shown in Figure S2) was used, and the outgoing neutron information was collected. The neutron absorption efficiency of scintillation screens of different thicknesses is shown in Figure 6c. The neutron absorption efficiency increased with the increase in the scintillation screen thickness and reached 90.1% when the thickness was 20 μm. Considering the range of internally converted electrons in the scintillation screen and the absorption efficiency of neutrons with different thicknesses of scintillation screens, a scintillation screen with a thickness of about 3μm has been selected in this paper. The GOS:Tb, 0.08F was mixed with polymer and coated on the substrate surface to form a cold neutron scintillation screen. The plane diagram of this scintillation screen is shown in Figure 6d. It can be seen that GOS:Tb, 0.08F particles were closely packed together with good surface flatness and no protruding particles. The scintillation screen profile was measured using a SEM (Figure 6e), and the thickness of the prepared scintillation screen was 2.5 μm. The resolution of the scintillation screen was tested on the cold neutron imaging system using the star template, and the results were recorded (see Figure 6f). Using a gadolinium Siemens star (Diameter: 20 mm, number of spokes: 128, the linewidths of radial markers: 4.5 μm~250 μm) as a resolution template, key technical indexes, such as the ultimate resolution of the scintillation screen in a cold neutron radiographic imaging device, were tested. Through image processing, the resolution reached about 12 μm. Limited by the whole neutron imaging system, the scintillation screen with a thickness of 2.5 μm could only reach the current resolution.

## 3. Materials and Methods

A high-temperature solid phase method was utilized in the GOS:Tb, F synthesis. Gadolinium oxide (Gd_2_O_3_; 99.99%, Aladdin), sublimed sulfur (S; 99.99%, Aladdin), terbium oxide sublimed sulfur (Tb_4_O_7_; 99.99%, Aladdin), sodium fluoride (NaF; 99.99%, Aladdin) and sodium carbonate (Na_2_CO_3_; A. R. grade, Sinopharm Chemical Reagent Co., Ltd., Shanghai, China) were used as received.

Synthesis of GOS:Tb, F scintillators: Firstly, Gd_2_O_3_, S, Tb_4_O_7_, Na_2_CO_3_ and NaF were dispensed on the basis of the stoichiometric ratio. They were transferred to an agate mortar, then mixed well and ground for 15 min. Subsequently, the mixture was placed in a crucible under the reducing atmosphere (8 vol% H_2_/92 vol% Ar) of a tube furnace. The sample was heated to 900 °C in the tube furnace for 4 h and then cooled to room temperature. Finally, the sample was washed to remove possible byproducts during the preparation process, and subsequently dried at a temperature of 80 °C. All GOS:Tb, F samples were prepared with 0.14 mole terbium/mole scintillators calculated by considering a total conversion of Gd_2_O_3_ into the corresponding Gd_2_O_2_S. The F doping concentrations were varied, such that the theoretical doping concentrations relative to Gd_2_O_3_ were 0, 0.01, 0.04, 0.08, 0.15 and 0.3 mol, respectively.

Preparation of GOS:Tb, F scintillator screens: The GOS:Tb, F scintillators were added to the binder solution (polyvinyl butyral (PVB, 2 wt%), n-butanol (98 wt%)), and stirred at high speed for 10 min to avoid the agglomeration of the scintillator and the PVB. The solution was then coated on the substrate surface with spin coating, with silicon as the substrate. After drying at room temperature, the GOS:Tb, F scintillation screen was prepared.

Characterization: XRD measurements were performed using an X-ray diffractometer (XRD, Bruker, Germany) with a step width of 0.02° and operated at 40 kV and 40 mA. The sample morphology was analyzed with a scanning electron microscope (SEM, S-4800, Tokyo, Japan) equipped with an energy dispersive spectroscope (EDS). Elemental composition was characterized by utilizing X-ray photoelectron spectroscopy (XPS, Thermo Scientific, Waltham, MA, USA). The transmission electron microscopy (TEM, Tokyo, Japan) and selected area electron diffraction (SAED) patterns were obtained using a JEM-2100F transmission electron microscope. The steady-state photoluminescence measurement was obtained using a fluorescence spectrometer (QY-2000, Hongkong, China). The fluorescence quantum yields were obtained with a fluorescence spectrometer (QY-2000, China) excited at 293 nm using a xenon lamp. The fluorescence decay of GOS:Tb, F was recorded with an ultra-fast lifetime Spectrofluorometer (DeltaFlex, Paris, France). The cold neutron tests [12] were carried out at the China Mianyang Research Reactor (CMRR). The conditions for the cold neutron source were as follows: the flux was about 5·10^6^ n/s/cm^2^, and the neutron wavelength was about 0.28 nm [39]. Simulations of the neutron absorption coefficients of GOS:Tb scintillation screens at different energies, and the neutron absorption efficiency at different thicknesses, were carried out using Geant4 software version 10.02 on an Intel i7-117008 CPU @ 2.5 GHz computer. The number of neutrons emitted in the simulation was 2 × 10^7^ in order to minimize the calculation errors and keep the calculation time as short as possible. The physical model of the simulation is shown in Figure S1, where the physical model was FTFP_BERT_HP (playing around with the “SetUseOnlyPhotoEvaporation(true)” of the particleHP module), the neutron source size was 2 cm × 2 cm, the scintillation screen size was 2 cm × 2 cm, the scintillation screen substrate was 2 cm × 2 cm × 200 μm of Si, and the neutron source was located 2 cm from the screen. The thickness of the scintillation screen was 0–50 μm when calculating the neutron absorption efficiency. The neutron energy is shown in Figure S2. If the neutron is not emitted from the scintillation screen, this indicated that it had been absorbed by the screen. The number of neutrons emitted from the scintillation was counted, which in turn allowed us to calculate the absorption coefficient of the scintillation screen. The energy spectrum of the internal conversion electrons was acquired by counting the energy information of the internal conversion electrons generated by the reaction of Gd with neutrons in the scintillation screen.

## 4. Conclusions

In summary, using a high-temperature solid phase method, highly efficient GOS:Tb, F green-emitting scintillators were prepared for use in neutron radiographs. The composition-dependent optical properties of both undoped and F-doped GOS:Tb were carefully studied. The results show that 8 mol% was the optimal doping level of F, that the GOS:Tb, F scintillator exhibited the best fluorescence intensity at 544 nm and that its QY can reach 77.21%. The thickness of the scintillation screen we prepared based on these findings was 2.5 μm, and the GOS:Tb, F scintillators were uniformly distributed on the whole substrate. This lays a good foundation for high-resolution imaging. Moreover, The GOS:Tb, F-based scintillation screen exhibited an outstanding neutron radiograph ability, with a spatial resolution of 12 μm. These results indicate the potential of GOS:Tb, F green-emitting scintillators with such admirable luminescence performance for use as high-resolution cold scintillation screens in neutron radiography.

## Figures and Tables

**Table 1:** Luminescence decay lifetime of the GOS:Tb, *x*F scintillators as a function of *x*.

*X*	0	0.01	0.04	0.08	0.15	0.3
Lifetime/μs	638	598	590	584	586	592

## Data Availability

Not applicable.

## Supplementary Materials

The following supporting information can be downloaded at: https://www.mdpi.com/article/10.3390/molecules28041815/s1, Figure S1: the simulation model of GOS:Tb scintillator; Figure S2: Incident cold neutron spectrum; Table S1: The elemental mass ratio of GOS:Tb.

## Sample Availability

Samples of the Gd_2_O_2_S:Tb, F scintillators are available from the authors.
