# Supplementary material for: High Quantum Efficiency Rare-Earth-Doped Gd2O2S:Tb, F Scintillators for Cold Neutron Imaging
Source: Molecules. 2023 Feb 15;28(4):1815. doi: 10.3390/molecules28041815 (PMC9959274; doi:10.3390/molecules28041815)
Supplement: Supplementary file 1 [file molecules-28-01815-s001.zip › molecules-2162921-supplementary.pdf]

High Quantum Efficiency Rare-Earth-Doped Gd_2O_2S:Tb, F Scintillators for Cold Neutron Imaging

Bin Tang [1], Wei Yin [1], Qibiao Wang [1,2,*], Long Chen [3,*], Heyong Huo [1], Yang Wu [1], Hongchao Yang [2], Chenghua Sun [3,*] and Shuyun Zhou [3]

[1] Institute of Nuclear Physics and Chemistry, China Academy of Engineering Physics, Mianyang, 621000, China; tangbin_e@163.com (B.T.); yinwei-itm@163.com (W.Y.); huoheyong@163.com (H.H.); nuclearwyang@163.com (Y.W.)

[2] School of Computer Science and Engineering, Sichuan University of Science & Engineering, Zigong, 643000, China; 18081478196@163.com

[3] Key Laboratory of Photochemical Conversion and Optoelectronic Materials, Technical Institute of Physics and Chemistry, Chinese Academy of Sciences, Beijing, 100190, China; zhou_shuyun@mail.ipc.ac.cn

* Correspondence: wangqibiao@suse.edu.cn (Q.W.); chenlong@mail.ipc.ac.cn (L.C.); sunchenghua@mail.ipc.ac.cn (C.S.)

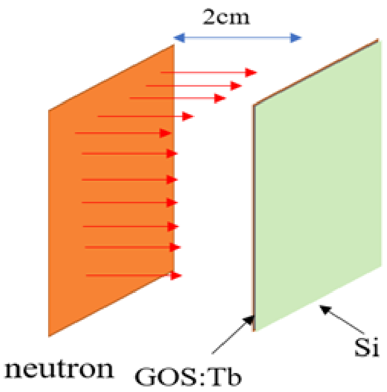

Figure. S1 the simulation model of GOS:Tb scintillator.

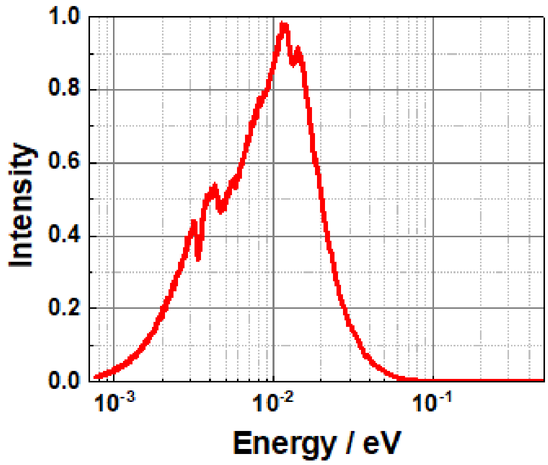

Fig. S2 Incident cold neutron spectrum.

Table S1. The elemental mass ratio of GOS:Tb.

Element	Gd	O	Tb	S
Mass ratio of element (%)	77.23	5.87	8.45	8.45
